# Supplementary material for: MetaRibo-Seq measures translation in microbiomes
Source: Nat Commun. 2020 Jun 29;11:3268. doi: 10.1038/s41467-020-17081-z (PMC7324362; doi:10.1038/s41467-020-17081-z)
Supplement: Supplementary file 10 — Supplementary Data 7 [file 41467_2020_17081_MOESM10_ESM.zip › File2/Confidence_High_Taxonomy/109474_out.krona.html]

Javascript must be enabled to view this page.

members
magnitude
magnitudeUnassigned
count
unassigned
taxon
rank

109474\_out

5

5
superkingdom
2

976
phylum
5

200643
class
5

5
171549
order

family
171552
5

genus
838
5


SRS013521\_contig\_number\_10044SRS049959\_contig\_number\_2971SRS078176\_contig\_number\_2086SRS1041031\_contig\_number\_29622SRS1041140\_contig\_number\_21559
165179
species
5
